# Modelling motor units in 3D: influence on muscle contraction and joint force via a proof of concept simulation

**DOI:** 10.1007/s10237-022-01666-2

**Published:** 2022-12-27

**Authors:** Harnoor Saini, Thomas Klotz, Oliver Röhrle

**Affiliations:** 1 Institute of Modelling and Simulation of Biomechanical Systems, University of Stuttgart, Pfaffenwaldring 5a, 70569 Stuttgart, BW Germany; 2 Stuttgart Center for Simulation Technology (SC SimTech), University of Stuttgart, Pfaffenwaldring 5a, 70569 Stuttgart, BW Germany

**Keywords:** Motor unit, Finite element, Continuum mechanics, Skeletal muscle, Functional heterogeneity, Task dependence

## Abstract

Functional heterogeneity is a skeletal muscle’s ability to generate diverse force vectors through localised motor unit (MU) recruitment. Existing 3D macroscopic continuum-mechanical finite element (FE) muscle models neglect MU anatomy and recruit muscle volume simultaneously, making them unsuitable for studying functional heterogeneity. Here, we develop a method to incorporate MU anatomy and information in 3D models. Virtual fibres in the muscle are grouped into MUs via a novel “virtual innervation” technique, which can control the units’ size, shape, position, and overlap. The discrete MU anatomy is then mapped to the FE mesh via statistical averaging, resulting in a volumetric MU distribution. Mesh dependency is investigated using a 2D idealised model and revealed that the amount of MU overlap is inversely proportional to mesh dependency. Simultaneous recruitment of a MU’s volume implies that action potentials (AP) propagate instantaneously. A 3D idealised model is used to verify this assumption, revealing that neglecting AP propagation results in a slightly less-steady force, advanced in time by approximately 20 ms, at the tendons. Lastly, the method is applied to a 3D, anatomically realistic model of the masticatory system to demonstrate the functional heterogeneity of masseter muscles in producing bite force. We found that the MU anatomy significantly affected bite force direction compared to bite force magnitude. MU position was much more efficacious in bringing about bite force changes than MU overlap. These results highlight the relevance of MU anatomy to muscle function and joint force, particularly for muscles with complex neuromuscular architecture.

**Supplementary Information:**

The online version contains supplementary material available at 10.1007/s10237-022-01666-2.

## Introduction

The motor unit is the fundamental element of force production in the neuromuscular system, comprising all muscle fibres that are innervated by a single motor neuron (Sherrington 1929). A motor unit is typically classified by the number of fibres that belong to it (size), and the contractile properties of those fibres. For example, type-S, -FR, and -FF motor units comprise, type I, type IIa, and IIb fibres, respectively. The number of recruited motor units together with their rate of stimulation determines the force exerted by a muscle (Adrian and Bronk 1929). In general, motor unit recruitment follows Henneman’s size principle (Henneman 1957), although it can be altered by the type of task, contraction velocity, or sensory feedback (Schindler et al. 2014; Ogawa et al. 2006; Romaiguére et al. 1989; Hodson-Tole and Wakeling 2009).

Besides varying in size, motor units also show different anatomy. Motor unit anatomy refers to the distribution of its fibres across the muscle and the cross-sectional area enclosing these fibres refers to its territory. While muscle anatomy varies greatly both within and between individuals, motor unit territories show common features such as size variation, overlap with other territories, and irregular shape (Bodine-Fowler et al. 1990; Roy et al. 1995, 1995). Additionally, motor unit types may be restricted to certain parts of the muscle cross-sectional area (Mesin et al. 2010; Tonndorf and Hannam 1994). The deep portions of the tibialis anterior, for example, have a higher proportion of type-FF and -FR motor units (Henriksson-Larsén et al. 1985; Mesin et al. 2010).

The interplay between the selective recruitment of motor units and their anatomy can lead to heterogeneous muscle contraction. This phenomenon was described by Herring et al. as “a single muscle’s capacity to create a multitude of force vectors through selective localised contraction” (Herring et al. 1979). The temporalis and masseter motor unit, for example, are selectively recruited depending on bite force direction (Blanksma et al. 1990; Ogawa et al. 2006). (Blanksma et al. 1990) used fine-wire electrodes to measure activity in different regions of the temporalis and found that, apart from the anterior regions, the muscle showed alterations in activity in response to changes in bite force direction. (Phanachet et al. 2003) used fine-wire electrodes to record electromyography signals of single motor unit at various locations in lateral pterygoid and found that 14 % of motor units were specialised to one task. Similarly, (Schindler et al. 2014) used fine-wire electrodes in the masseter and found that about half (46 %) of the investigated motor unit were specialised to one task. (Staudenmann et al. 2009) used a grid of surface electromyography electrodes to record the activity of the triceps surae during plantar flexions along predefined directions while recording forces at the foot and showed correlations between different surface activity and the directions of recorded force. Investigating the same muscle (group), (Wakeling 2009) used a coarser surface electromyography grid to measure muscle activity during cycling against different resistances and showed very little correlation within regions of the same muscle, implying selective recruitment of the muscle regions. These studies, among others (ter et al. 1984; Pérot et al. 1996; Turkawski et al. 1998; Guzmán et al. 2015; Staudenmann et al. 2009; Murray et al. 1999; Blanksma et al. 1955), suggest that the task dependence of skeletal muscles is the norm rather than the exception.

Human movement is often investigated by measuring the body’s kinematics and estimating corresponding muscle activations by applying inverse-dynamics to biomechanical models (e.g. Lloyd and Besier 2003; Pizzolato et al. 2017; Harandi et al. 2020). Typically, such models are based on multi-body simulations, which represent muscles as lumped contractile units, i.e. they neglect the functional organisation of skeletal muscles into motor units. At most, only statistical information about motor unit firing is used (e.g. Sartori et al. 2017). Therefore, the interplay between motor unit anatomy and human movement remains largely unexplored. In contrast, 3D, macroscopic, continuum-mechanical muscle models (3D models) do indeed have the ability to spatially resolve motor units. However, such models have largely ignored this feature of muscle contraction (e.g. Johansson et al. 2000; Fernandez et al. 2005; Blemker and Delp 2005; Röhrle and Pullan 2007; Wu et al. 2014; Röhrle et al. 2017; Weickenmeier et al. 2017; Chi et al. 2010; Péan et al. 2019)—most likely due to the representation of muscle fibres as a continuous vector field, which makes the grouping of fibres to define motor unit anatomy challenging. Currently, only multi-scale continuum-mechanical muscle models resolve motor unit anatomy by describing individual muscle fibres (e.g. Heidlauf and Röhrle 2014; Schmid et al. 2019; Röhrle et al. 2012; Marcucci et al. 2017; Teklemariam et al. 2019). However, since these multi-scale models are computationally very expensive to solve, existing simulation studies have been restricted to idealised, isolated muscles or fibre bundles.

Within this work, we propose an approach that combines both motor unit activity and anatomy into 3D models to represent the heterogeneous nature of muscle contraction. We do this by decomposing muscle activity into motor unit activity and anatomy, resulting in a spatiotemporal muscle activation parameter. This requires the computation of physiologically plausible motor unit distributions, activity, and a method to incorporate this information into a macroscopic continuum-mechanical framework. First, a brief overview of continuum-mechanical theory and constitutive modelling is provided (Sect. 2.1). Followed by the methods for generating motor unit information. Motor unit anatomy is created via a novel “virtual innervation” technique (Sect. 2.2.1), while motor unit activity models are drawn from the literature (Sect. 2.2.2). Discrete motor unit anatomy, on the muscle fibre scale, is then incorporated into the continuum mechanical, finite element framework, requiring a mapping of the (microscale) motor unit properties to the (macroscopic) finite element mesh and an assumption on action potential conduction velocity. An idealised 2D geometry is used to investigate the mesh dependency of muscle activity computation (Sect. 2.3.1) and an idealised 3D geometry to verify the influence of instantaneous action potential propagation on force production (Sect. 2.3.2). Finally, we demonstrate virtual innervation on the masseters of a masticatory model and generate a range of unique motor unit distributions. Each unique masseter is activated by an identical motor unit recruitment protocol to simulate a static bite. The differences in the bite force reveal the interplay between motor unit distribution and muscle function (Sect. 2.3.3). Our technique for creating motor unit-enriched 3D models provides a transparent environment for studying relationships between motor unit physiology, muscle function, and movement, such as muscle task dependency.

## Methods

### Three-dimensional musculotendon modelling

Skeletal muscles undergo large deformations during contraction and movement, making the theory of finite elasticity a suitable choice to describe their mechanical response. Here, we provide a very brief sketch of the theory. For further details, see Holzapfel (2000); Bonet and Wood (1997) for example.

Consider the musculotendon complex as a continuum body ${\mathcal {B}}_0$ (at $t=0$, ${\mathcal {B}}$ otherwise) comprised of material points ${\mathcal {P}}$. Each material point ${\mathcal {P}}$ has coordinates $\varvec{X}$ in the reference configuration ($t=0$), being mapped to $\varvec{x}$ in the current configuration (at time *t*). This mapping is described by the placement function $\varvec{x}=\varvec{\chi }(\varvec{X},t)$. Then, the deformation and motion of each material point ${\mathcal {P}}$ is described by the deformation gradient tensor $\varvec{F}=\partial {\varvec{x}}/\partial {\varvec{X}}$, and the right Cauchy–Green deformation tensor $\varvec{C} = \varvec{F}^{\text {T}}\!\varvec{F}$ provides a rotation invariant deformation measure.

The balance of linear momentum governs the deformation of ${\mathcal {B}}$ and is given here in its local form with respect to the current configuration, i.e. 

1 $$\begin{aligned} \mathrm{div}\varvec{\sigma }= \rho \ddot{\varvec{u}}, \end{aligned}$$

where $\varvec{u}=\varvec{x}-\varvec{X}$ is the displacement vector in m, $\rho$ is the mass density in kg/m$^3$ and $\varvec{\sigma }$ is the Cauchy stress tensor in N/m$^2$. Body forces, such as gravity, are absent in the momentum balance as we assume them to be negligible compared to muscle forces.

*Constitutive modelling* The stress in Eq. 1 describes the material’s mechanical behaviour based on its constitutive relation. Details of the particular constitutive relation used to describe the musculotendon complex are given in Saini and Röhrle (2022); Röhrle et al. (2017) and are only briefly covered here. We assume that skeletal muscle is hyperelastic, transversely isotropic, and nearly incompressible. Furthermore, the passive and active stresses are additively decomposed, i.e. $\varvec{S}=\varvec{S}_{\text {pass}}+\varvec{S}_{\text {act}}$, where $\varvec{S}$ is the second Piola–Kirchhoff stress tensor. The passive stress is further decomposed between contributions from the (isotropic) bulk and along the (anisotropic) fibre direction, i.e. $\varvec{S}_{\text {pass}}=\varvec{S}_{\text {iso}}+\varvec{S}_{\text {aniso}}$.

The passive stresses are derived from strain energies, with $\varvec{S}_{\text {iso}}$ and $\varvec{S}_{\text {aniso}}$ characterised by Mooney–Rivlin (Mooney 1940; Rivlin and Rideal 1948) and Balzani et al. (206) formulations, respectively. Accordingly, the isotropic stress is described by

2 $$\begin{aligned} \varvec{S}_{\text {iso}} = 2 \, \left[ \left( c_1 + I_1 c_2\right) \, \varvec{I}- c_2 \, \varvec{C}+ \left( cJ(J-1)-\frac{d}{2} \right) \, \varvec{C}^{-1} \right] , \end{aligned}$$

and the anisotropic stress by

3 $$\begin{aligned} \varvec{S}_{\text {ansio}}= {\left\{ \begin{array}{ll} \displaystyle 2\, c_3 \, c_4 \, \, \varvec{M} \left( I_4-1 \right) ^{c_4-1} + 2\,c_5 \, c_6 \, \varvec{M} \, \left( I_4-1\right) ^{c_6-1} \quad &{}\text {if } I_4\ge 1,\\ 0 &{}\text {otherwise.} \end{array}\right. } \end{aligned}$$

In Eqs. 2 and 3, $c_{1-6}$ are independent material parameters, all in N/m$^2$ except for $c_4$ and $c_6$, which are dimensionless. The parameter $d=2(c_1+2\,c_2)$ ensures a stress-free reference configuration, and *c* penalises dilatational (volume changing) deformations. The Jacobian $J=\text {det}({\varvec{F}})$ deviates from unity under such dilatational deformations. The invariants of $\varvec{C}$ are defined by $I_1=\text {tr}(\varvec{C})$ and $I_4=\text {tr}(\varvec{M}\varvec{C})=\lambda _{\text {f}}^2$, where $\lambda _{\text {f}}$ is the fibre stretch. Lastly, $\varvec{M}=\varvec{a}_0\otimes \varvec{a}_0$ is a structural tensor, which describes the (transverse) anisotropy of the material via the fibre direction in the reference configuration $\varvec{a}_0$.

The active stress is described by

4 $$\begin{aligned} \varvec{S}_{\text {act}}= \frac{S_{\text {max}}}{I_4} \, \alpha _{\text {m}} \, f_{\text {l}}(\lambda _{\text {f}}) \, \varvec{M}, \end{aligned}$$

where $S_{\text {max}}$ is the peak (isometric) stress that the muscle can produce, in N/m$^2$, and $f_{\text {l}}\in [0,1]$ represents the normalised amount of (actin and myosin) filament overlap, i.e. the force-length relationship of muscle. It is characterised by

5 $$\begin{aligned} f_{\text {l}}(\lambda _{\text {f}}) = \exp \left\| \dfrac{ \frac{ \lambda _{\text {f}} }{ \lambda _{\text {f}}^{\text {opt}} }-1 }{ w_i }\right\| ^{v_i}, \end{aligned}$$

where $\lambda ^{\text {opt}}_{\text {f}}$ is the optimal fibre length at which $S_{\text {max}}$ is produced. The parameters $\omega _i$ and $v_i$ determine the shape of the force-length curve, where the subscripts $i=\text {asc}$ and $i=\text {dsc}$ when $\lambda _{\text {f}} < \lambda _{\text {f}}^{\text {opt}}$ and $\lambda _{\text {f}} > \lambda _{\text {f}}^{\text {opt}}$, representing the ascending and descending branches of the force-length relationship, respectively.

Lastly, $\alpha _{\text {m}} \in [0,1]$ represents the normalised muscle activity, varying between 0 and 1 for fully passive and active states, respectively. Traditionally this is only a temporally varying quantity, here we incorporate both (motor unit) temporal and spatial information to compute heterogeneous muscle activity. The computation of this motor unit information is described in Sect. 2.2.

The material-behaviour of the musculotendon complex also needs to account for connective tissues such as tendons and aponeuroses. This is achieved by generalising the muscle’s constitutive relation with a tissue parameter $\gamma \in [0,1]$, i.e. 

6 $$\begin{aligned} \varvec{S}^{\text {mtc}} = \varvec{S}_{\text {iso}}(\gamma ) + \varvec{S}_{\text {ansio}}(\gamma ) + \gamma \, \varvec{S}_{\text {act}}, \end{aligned}$$

where $\gamma$ varies between 0 for pure tendon and 1 for pure muscle. To capture the different material responses of the tissues, the material parameters are linearly interpolated between values characterising pure tendon $c_{i}^{\text {t}}$ and pure muscle $c_{i}^{\text {m}}$ by $c_{i}^{\text {mtc}}=(1-\gamma )\,c_{i}^{\text {t}}+\gamma \,c_{i}^{\text {m}}$, $i=\{1,\ldots ,6\}$ (Eqs. 2 and 3).

### Enriching muscle activity with motor unit information

By assuming that muscle activity $\alpha _{\text {m}}$ (Eq. 4) can be fully characterised by the activity and distribution of muscle fibres at the microscale, we decompose the activation parameter accordingly (e.g. Röhrle et al. 2019), That is, we assume that the properties of all muscle fibres belonging to the same motor unit are identical and that they are activated synchronously. Then, individual motor unit activity can be described by a single, normalised, time-dependent function: $\alpha _i(t) \in [0, 1]$ ($i=1,\ldots ,N_{\text {MU}}$, where $N_{\text {MU}}$ is the total number of motor unit). We further assume that the spatial distribution of a motor unit in the muscle can be described by a volume fraction ${\bar{\kappa }}_i(\varvec{x}) \in [0, 1]$ ($\varvec{x} \in {\mathcal {B}}$). Then, the activation of the muscle (at any point $\varvec{x}$ and time *t*) can be computed by the ensemble of motor unit activity and distribution:

7 $$\begin{aligned} \alpha _{\text {m}}(t,\varvec{x}) = \sum _{i=1}^{N_{\text {MU}}} \, \alpha _i(t) \, {\bar{\kappa }}_i(\varvec{x}). \end{aligned}$$

Both motor unit activities and distributions are biophysically and micromechanically derived and are discussed in Sects. 2.2.1 and 2.2.2, respectively. By decomposing muscle activity in this way, we assume that the entire region occupied by the motor unit is activated simultaneously, i.e. the influence of propagating action potential is neglected. We investigate the error in motor output this introduces by comparison to a multi-scale, multi-physics model in Sect. 2.3.2.

#### Motor unit anatomy via virtual innervation

The virtual innervation process can be summarised as follows: *(Step 1)* reconstruct fibre microstructure from the fibre orientation field. *(Step 2)* define the number of motor unit and their size. *(Step 3)* perform virtual innervation iteratively to group the discrete fibres into motor unit. *(Step 4)* homogenise the innervated fibres within the finite element mesh.

*(Step 1)* Muscle architecture is represented via a fibre orientation field $\varvec{a}_0(\varvec{X})$ ($\varvec{X}\in {\mathcal {B}}$). We reconstruct idealised representations of the muscle microstructure (“fibre scaffolds”) using an explicit streamline tracking method (e.g. Kupczik et al. 2015). This ensures that the reconstructed fibre scaffolds ${\mathcal {F}}^k$ ($k=1,\ldots ,N_{\text {SC}}$, where $N_{\text {SC}}$ is the total number of scaffolds) are compatible with the idealised continuous description of the muscle fibre architecture, i.e. the vector field describing the muscle fibre direction. A discrete representation of the scaffolds, i.e. given by a set of points $\varvec{X}_{\text {micro}}^k \, \in \, {\mathcal {F}}^k$, is iteratively computed by

8 $$\begin{aligned} \varvec{X}_{\text {micro}}^k(r+1) = \varvec{X}_{\text {micro}}^k(r) + \varvec{a}_0(\varvec{X}_{\text {micro}}^k(r)) \, \Delta _f, \end{aligned}$$

where *r* parametrises the scaffold, $\varvec{a}_0(\varvec{X}_{\text {micro}}^k(r))$ is the fibre-orientation at the previous scaffold coordinate, and $\Delta _f$ is the step size along the fibre scaffold. Fibre scaffold tracking is initiated from a seed point $\varvec{s}^k$ ($k=1,\ldots ,N_{\text {SC}}$). The seed points are ensured to lie along the middle of the muscle geometry (retroactively). The tracking process yields a set of scaffolds ${\mathcal {F}}^k$ with a corresponding set of seed points: ${\mathcal {S}}= \{\varvec{s}^k \}$.

*(Step 2)* The number of motor units to assign are specified by $N_{\text {MU}}$. Motor unit sizes are characterised by their innervation ratio, which is the number of fibre scaffolds they contain. An exponential distribution of innervation ratios is used, based on Enoka and Fuglevand (2001), i.e. 

9 $$\begin{aligned} \text {IR}_{\text {s},i} = \left\lceil N_{\text {SC}}\,\frac{\text {IR}_1}{\text {IR}_{\text {tot}}} \, \exp \left( i \, \frac{\ln r_{\text {IR}}}{N_{\text {MU}}} \right) \right\rceil , \quad i=1,\ldots ,N_{\text {MU}}. \end{aligned}$$

Here, $r_{\text {IR}}=\text {IR}_{N_{\text {MU}}}/\text {IR}_1$ is the ratio between the innervation ratio of the largest and smallest motor unit, IR$_{\text {tot}}=\sum _i^{N_{\text {MU}}}\text {IR}_i$ is the total number of fibres in the muscle, and $\lceil \cdot \rceil$ is the ceiling function.

*(Step 3)* The virtual innervation can be considered as gathering $N_{\text {SC}}$ fibre scaffolds into $i=\{1,\ldots ,N_{\text {MU}}\}$ groups, with the number of scaffolds per group defined by $\text {IR}_{\text {s},i}$. Here, we introduce the subscript $(\cdot )_i$, which denotes that a quantity belongs to motor unit *i*, e.g. ${\mathcal {F}}^k \rightarrow {\mathcal {F}}^k_i$. This grouping process, or virtual innervation, proceeds iteratively from the smallest to the largest motor unit, i.e. first $\text {IR}_{\text {s},1}$ fibres are grouped, then $\text {IR}_{\text {s},2}$, and so on, until $\text {IR}_{\text {s},N_{\text {MU}}}$. A fibre scaffold (or seed point) can only be innervated by a single motor unit. First, the axon of a given motor unit *i* innervates a random seed point and this is set as the so-called reference seed point ($\varvec{s}^k \rightarrow \varvec{s}^k_i = \varvec{c}_i$). Second, the remaining ($\text {IR}_{\text {s},i}-1$) axons continue to (randomly) innervate “free” seed points about the reference seed point $\varvec{c}_i$, within a distance *R*, i.e. 

10 $$\begin{aligned} f_{\text {d}}({\mathcal {S}}^{\text {u}},\varvec{c}_i) < R, \end{aligned}$$

where $f_{\text {d}}$ is a distance metric (discussed below) and ${\mathcal {S}}^{\text {u}}$ is introduced as the set of currently uninnervated seed points. Once the desired number of seed points $\text {IR}_{\text {s},i}$ have been innervated, the axon for motor neuron $i+1$ innervates another (uninnervated) seed point and a new reference seed point is set ($\varvec{c}_{i+1}$). Then, the remaining axons of motor neuron $i+1$ subsequently seek out free seed points about $\varvec{c}_{i+1}$ (according to Eq. 10). The process continues for all remaining motor neurons. When a seed point is innervated, i.e. $\varvec{s}^k\rightarrow \varvec{s}^k_i$, the corresponding fibre scaffold including its microstructural points are also innervated, i.e. $\varvec{X}_{\text {micro}}^k \rightarrow \varvec{X}_{\text {micro},i}^{k}$.

The search space for the reference seed point selection is restricted to a subregion of the muscle’s cross section. This restriction is achieved by setting a seed point as an anchor point $\varvec{c}_{\text {a}}$, and limiting the reference seed point search within a distance *D* about the anchor point, i.e. 

11 $$\begin{aligned} f_{\text {d}}({\mathcal {S}}^{\text {u}},\varvec{c}_{\text {a}}) < D. \end{aligned}$$

This is analogous to Eq. 10. However, while *R* restricts the innervation of the axons of a given motor unit (about the reference seed point), *D* restricts the innervation of the reference seed points themselves (about the anchor point). The anchor point $\varvec{c}_{\text {a}}$ remains fixed for the entire innervation process. Note that it may be the case that as the innervation proceeds, no free seed points can be found within distances *R* and *D*. When this occurs, the selection distances *R* and *D* are incremented by $\Delta R$ and $\Delta D$, and the searches are repeated.

The distance measure used to perform the search for (free) seed points is the generalised ellipsoidal distance, i.e. 

12 $$\begin{aligned} f_{\text {d}}(\varvec{a},\varvec{b})^2 = (\varvec{a}-\varvec{b}) \; \varvec{W} \; (\varvec{a}-\varvec{b})^{\text {T}}, \end{aligned}$$

where $\varvec{W}$ is a weighting matrix which acts to scale the distance measure along its eigenvectors $\varvec{w}_1,\varvec{w}_2,\varvec{w}_3$ by the amount given by its eigenvalues, i.e. by $\lambda _1,\lambda _2,\lambda _3$. For example, $\varvec{W}=\varvec{I}$ (identity matrix) gives the Euclidean distance. In summary, the parameters $\{\varvec{c}_{\text {a}},R,D,\Delta R, \Delta D, \varvec{W}\}$ characterise the virtual innervation procedure.

*(Step 4)* Thus far, motor unit anatomy has been described via discrete microstructural points. To map the innervated microstructure to the continuum-mechanical model, a statistical approach is used (e.g. Beran 1968; Kröner 1972). For a point in the muscle ${\mathcal {P}}\in {\mathcal {B}}_0$ with the coordinates $\varvec{X}$, a nearest neighbour search is used to group all fibre scaffold microstructural points $\varvec{X}_{\text {micro},i}^k \ (k=1,\ldots ,N_{\text {SC}})$ into (virtual) tissue samples. These samples are considered as statistical volume elements. Then, motor unit volume fractions ${\bar{\kappa }}_i(\varvec{X}) \ (i=1,\ldots ,N_{\text {MU}})$ are computed as the probability of finding a fibre scaffold point associated with motor unit *i* in the given statistical volume element, i.e. 

13 $$\begin{aligned} {{\bar{\kappa }}_i(\varvec{X})=\frac{N_{\text {micro},i}(\varvec{X})}{N_\text {micro}(\varvec{X})}}, \end{aligned}$$

where $N_\text {micro}$ is the number of microstructural points that have $\varvec{X}$ as their nearest neighbour, of those $N_{\text {micro},i}$ are the number of microstructural points that belong to motor unit *i*. Note that the motor unit volumes are “etched” into the muscle and are transformed to the current configuration via the placement function, i.e. ${\bar{\kappa }}_i(\varvec{X})={\bar{\kappa }}_i(\varvec{\chi }(\varvec{X},t))$.

#### Motor unit activity

With the motor unit distributions at hand, we compute their activity for the duration of the simulation, $t \in [0,T]$. We assume that contractile properties and activity of a motor unit’s fibres are identical. Therefore, a representative muscle fibre is used to characterise each motor unit's activity level. Recruitment and excitation-contraction coupling of the representative fibres is summarised as follows: *(Step 1)* A phenomenological model (Fuglevand et al. 1993) predicts motor neuron discharge times in response to volitional muscle excitation *E*(*t*). *(Step 2)* These discharges are coupled with a phenomenological membrane model (Aliev and Panfilov 1996) and *(Step 3)* a cross-bridge dynamics model (Razumova et al. 1999) simulates excitation-contraction coupling in the representative muscle fibre (Heidlauf et al. 2017, see also). This yields motor unit activities $\alpha _i(t)$, which are unidirectionally coupled to the continuum-mechanical skeletal muscle model. That is, $\alpha _i(t)$ enters Eq. 7 and together with the motor unit fraction ${\bar{\kappa }}_i(\varvec{X})$ determines the local muscle activation $\alpha _\mathrm {m}(t,\varvec{x})$, which, in turn, enters Eq. 4. In the following, we only briefly summarise the recruitment-, calcium- and cross-bridge models; further details can be found in the literature, i.e. (Fuglevand et al. 1993; Razumova et al. 1999; Aliev and Panfilov 1996; Heidlauf et al. 2017).

*(Step 1)* The motor neuron discharge times $t_{i,p}$, i.e. denoting the *p*th firing time of motor neuron *i*, are computed by supplying a global excitatory drive *E*(*t*) to the motor neuron-pool. A motor neuron fires only when *E*(*t*) has exceeded its recruitment-threshold $R_i$. The recruitment-thresholds are taken as proportional to the size of the motor unit (in terms of its innervation ratio) according to the size principle (Henneman 1957) (Fuglevand et al. 1993, c.f.). The mean frequency at which a motor neuron *i* fires is determined by

14 $$\begin{aligned} {\bar{f}}^{\text {r}}_i(t) = E(t)-R_i + f^{\text {m}} \quad \text {when} \quad E(t) > R_i \quad \text {and} \quad {\bar{f}}^{\text {r}}_i(t) \le f^{\text {p}}_i, \end{aligned}$$

where $f^{\text {p}}_i$ and $f^{\text {m}}$ are the peak and minimum firing rates in Hz, respectively.

*(Step 2)* The discharge of a motor neuron gives rise to an action potential in the muscle fibre, which triggers the release of calcium (Ca$^{2+}$) into the sarcoplasm. This leads to cellular force production via cross-bridge cycling. In the phenomenological two-state membrane model of Aliev and Panfilov (1996), the fast-state variable represents the transmembrane potential. Further, it is assumed that the model’s slow variable behaves qualitatively similar to the intracellular Ca$^{2+}$concentration (e.g. Heidlauf et al. 2017).

*(Step 3)* Controlled by the Ca$^{2+}$concentration, a simplified Huxley-type model (Razumova et al. 1999) simulates the muscle fibre’s force output. Briefly, the model represents cross-bridges in four distinct states; with state transitions modelled by first-order kinetics. Although not described here, the rate coefficients dictate the twitch properties of the motor unit (or representative fibre). Here, rather than distinct categories of slow- and fast-twitch motor unit, we linearly interpolated the rate coefficients to create a smooth transition between the slowest- and fastest-twitch motor unit. Lastly, motor unit (or representative fibre) activity is assumed to be represented by the fraction of cross-bridge in the post-power stroke state ($A_{2,i}$), i.e. 

15 $$\begin{aligned} \alpha _i(t) = \frac{A_{2,i}(t)}{A_{2,i}^{\text {max}}}, \quad i=1,\ldots ,N_{\text {MU}}, \end{aligned}$$

where $A_{2,i}^{\text {max}}$ is the maximum amount of post-power stroke cross-bridge in the representative fibre (of motor unit) *i*.

### Simulation trials

We used idealised geometries to investigate modelling assumptions prior to applying the method to an anatomically realistic musculoskeletal model as a proof-of-concept. Muscle activity is mesh dependent in the finite element mesh since it is computed by statistical averaging of a discrete representation of motor unit anatomy. We investigated this mesh dependency by using an idealised, 2D microstructural muscle model to compare “ground truth” activity (for various motor unit distributions) to that averaged at coarser mesh sizes (Sect. 2.3.1). Next, we analysed the impact of assuming instantaneous action potential propagation on force production of an idealised, 3D musculotendon complex by comparison to a multi-scale and multi-physics chemo-electro-mechanical skeletal muscle model (Sect. 2.3.2). Lastly, we used an anatomically realistic model of the masticatory system as a proof-of-concept to investigate the relationship between motor unit anatomy and bite force (Sect. 2.3.3)

#### Idealised geometry: mesh dependency of muscle activity

**Fig. 1:**
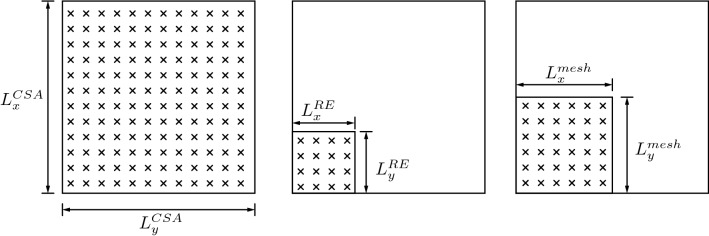
Ideal muscle geometry used for mesh dependency analysis. Each cross represents a virtual fibre, and the inter-fibre distance (or fibre diameter) is 100 µm. The reference element (RE) contains 4-by-4 fibres. Mesh sizes greater than RE sizes are considered, with an exemplary mesh size shown

Motor unit volume fractions ${\bar{\kappa }}_i(\varvec{X})$ are computed via statistical averaging within the finite element mesh (Eq. 13). As a result, motor units are no longer spatially resolved at the individual fibre level but rather are represented by continuous distributions that are approximated with the finite element method. Since muscle activity is determined by motor unit distribution and activity (i.e. Eq. 7), the solution depends on the discretisation of the finite element mesh. We investigated this mesh dependency via an idealised, 2D, muscle cross section (Fig. 1). The analysis is summarised as follows: *(Step 1)* generate an idealised model of muscle cross section with muscle fibres (micrometre scale).

*(Step 2)* Virtually innervate the fibres to form motor units with different distributions.

*(Step 3)* Define various element sizes and compute the muscle activity within each mesh element. The solution for the finest macroscopic discretisation, termed as the reference element (RE), is used as reference to compute the error of the muscle activity.

*(Step 1)* An idealised virtual muscle cross section $L_{\text {x}}^{\text {CSA}}=L_{\text {y}}^{\text {CSA}}=L^{\text {CSA}}=40\,\mathrm{mm}$ (cross-sectional area 16 cm$^2$, Fig. 1 left) was populated with evenly spaced fibres at a distance of $L_{\text {x}}^{\text {fib}}=L_{\text {y}}^{\text {fib}}=L^{\text {fib}}=100\,\mu\mathrm{m}$ (Feher 2012; Polgar et al. 1973), giving a total of 16,000 fibres. Additionally, a reference element (RE) denoting the finest discretisation was defined as containing 4-by-4 fibres, yielding a RE size of $L_{\text {x}}^{\text {RE}}=L_{\text {y}}^{\text {RE}}=L^{\text {RE}}=400\,\mu\mathrm{m}$ (Sharafi and Blemker 2011; Virgilio et al. 2015; Spyrou et al. 2017, 2019, c.f.) and totalling $N_{\text {RE}}={10000}$ REs (Fig. 1 middle).

*(Step 2)* Innervation of the virtual fibres was carried out according to the algorithm described in Sect. 2.2.1. The motor unit pool was characterised by $N_{\text {MU}}=50$, IR$_1=100$, and IR$_{\text {tot}}={10000}$. Three different motor unit distributions were considered: completely random (maximal overlap), medium overlap, and minimal overlap. The respective shape parameters were: $R=D=\Delta R=\Delta D$ equalling $2\,L^{\text {CSA}}$, $0.25\,L^{\text {CSA}}$, and $0.1\,L^{\text {CSA}}$. The parameters $\varvec{c}_{\text {a}}=[L^{\text {CSA}}/2, L^{\text {CSA}}/2]$ and $\varvec{W}=\varvec{I}$ were kept identical for all 3 distributions.

*(Step 3)* Statistical averaging was used to compute the motor unit volume fractions within each element for the different mesh sizes. With the volume fractions at hand, the muscle activity can be computed via Eq. 7. Different mesh sizes were defined in terms of the number of elements along a given side. Then the error in muscle activity was taken as the mean difference between RE and mesh activities, i.e.

16 $$\begin{aligned} \alpha ^{\text {error}}_{\text {mean},k} = \sum _{k=1}^{N_{\text {RE}}} \vert \alpha ^{\text {RE}}_k - \alpha ^{\text {mesh}}_e \vert , \end{aligned}$$

where $k=1,\ldots ,N_{\text {RE}}$ and the element *e* is found by a nearest neighbour search from RE *k*. Note that this error computation was repeated for each of the different mesh sizes considered. The mean per-mesh error was compared to mesh size for each of the 3 motor unit distributions considered.^1^

#### Idealised geometry: modelling assumptions

The details of the multi-scale and multi-physics model (CEM model) used to investigate the influence of action conduction velocity can be found in Röhrle et al. (2012); Heidlauf and Röhrle (2014). Briefly, the CEM model consists of 1D muscle fibres embedded in a continuum-mechanical skeletal muscle model, where the fibres act to transmit the action potential from the neuromuscular junction outwards. We used the model to compare two cases, one where action potentials propagate along the fibres, and another, where the action potentials arise instantly along the fibres. The latter case reflects the modelling assumption used in the current model, i.e. synchronised activity along and between all fibres of a motor unit. The simulations were performed on a single core (Intel Core i7-4790K @ 4 GHz) with 24 GB of memory.

An idealised cuboid geometry with dimensions $L\times W\times H$ of $40 cm\times 10 cm\times 10 cm$ of a musculotendon complex was used. Both muscle and tendon tissue had the same cross section, with a muscle-to-tendon ratio of 2.5. The muscle was populated with 16 fibres, which were grouped in 5 motor units. The innervation ratios of motor unit 1–5 were 2;2;2;4;5, respectively. Virtual innervation was carried out with parameters $D=R=\Delta R=\Delta D=W/4$, with $\varvec{W}=\varvec{I}$ and $\varvec{c}_{\text {a}}=[L/2, W/2, H/2]$.

Stimulation of the motor units followed fixed firing rates of 28;17;14;10;7Hz for motor units 1–5, respectively. The motor units were recruited sequentially with a 10 ms offset from the preceding motor unit. For the first case, a current pulse was injected into the central node of the respective fibres. In the second case, an identical stimulus was delivered to all nodes of the respective fibres simultaneously. We adapted all parameters, besides the geometry, from the baseline experiment in Schmid et al. (2019). To compare the mechanical response of the two cases, the time taken for the force to attain a given value, defined as the time to force, was computed. Furthermore, electromechanical delay is calculated for both conditions as the time difference between the (first) stimulus and the time at which the musculotendon complex force increases by 1% of the maximum (twitch) force (Mörl et al. 2012).

#### Anatomical geometry: bite force specificity of masseters

The masticatory system model consists of the mandible, associated dental structures, and the left and right masseters. We systematically varied the motor unit territory distributions to produce multiple prototype models—each with unique masseter motor unit distributions. Then, each model was supplied with the same motor unit activity, and the resulting molar bite forces were taken as markers of motor output. Additionally, we simulated a static bite following the status-quo approach by applying an (equivalent) spatially constant activity to the masseters.

The setup of the simulation is as follows: *(Step 1)* define masticatory model geometry and masseter motor unit properties. *(Step 2)* define the motor unit recruitment protocol. *(Step 3)* perform the static bite simulation. Lastly, motor unit anatomy was quantified via metrics outlined in Sect. 2.3.4.

*(Step 1)* Details of the masticatory model geometry and material properties are given in our previous publications (Röhrle and Pullan 2007; Saini et al. 2020; Saini and Röhrle 2022). Here, we focus on the definition of the masseter’s motor unit territory. A total of approximately $N_{\text {SC}}={1200}$ fibre scaffolds were reconstructed within each masseter. These fibre scaffolds were grouped into $N_{\text {MU}}={50}$ motor units, with the smallest motor unit containing $\text {IR}_{\text {s},1}=8$ scaffolds and the largest $\text {IR}_{\text {s},50}=53$ (Eq. 9). That is, the formation of motor units 1 and 50 requires the innervation of 8 and 53 fibre scaffolds in the masseter, respectively.

All distributions shared two common features: First, smaller motor units were preferentially located anteriorly in the deep-head of the masseter by the placement of $\varvec{c}_{\text {a}}$ (Eq. 11) (e.g. Tonndorf and Hannam 1994; Eriksson and Thornell 1983). Second, the shape parameters $\lambda _1=1,\lambda _2=\lambda _3=5$ were chosen such that ellipsoidal-shaped territories were formed with their main-axis aligned along the masseters’ posterior-anterior axis (Eq. 12) (e.g. Tonndorf and Hannam 1994; McMillan and Hannam 1991) The remaining distribution parameters $\{R,D,\Delta R, \Delta D\}$ were varied to produce $n=7$ masticatory models (Table 1).

Lastly, the twitch properties of the motor units were as follows: the time to peak and peak twitch force for the smallest motor unit (number 1) were 0.091 N and 123 ms, respectively. The largest motor unit (number 50) had a time to peak a factor 3 smaller and a peak twitch force a factor 35 larger than the smallest motor unit. The properties for the remaining motor units were interpolated between the two extremes.

**Table 1:** Motor unit anatomy parameters for the motor unit territory distribution variations in the masseter. Blanks refer to the parameters in distribution $n=1$

Dist. (n)	*R* (mm)	*D* (mm)	$\Delta R$ (mm)	$\Delta D$ (mm)
1	1	10	4	2
2	15			
3		100		
4			20	
5				60
6	15	300		
7	10	100		

**Fig. 2:**
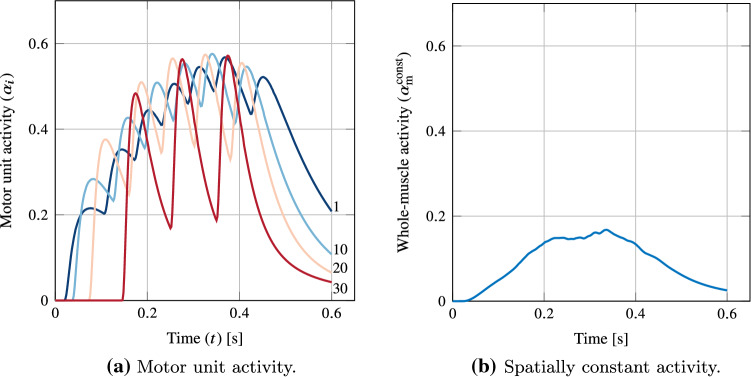
Activation protocols for the sub-maximal static bite for both **a** motor unit driven (motor unit numbers indicated) and **b** status-quo (spatially constant) approaches. Motor unit activity is a function of their firing times and twitch properties

*(Step 2)* The masseters were recruited via a ramped excitatory drive *E*(*t*). It was linearly increased from 0 to 0.375 over $\Delta t=0.25 s$, held constant for $\Delta t=0.1\,s$, and then decreased linearly to 0 over $\Delta t=0.25\,s$. At its peak, the excitatory drive recruited 34 out of 50 of the motor units in the pool (selected motor unit activities are shown in 2a). The resulting motor neuron firing rates were characterised by Eq. 14. The maximum motor unit firing rate and range of firing rates ($f^{\text {p}}_1$ and $f^{\text {D}}$, respectively) were 34Hz to 64 Hz (Kwa et al. 1995, 2002), the minimum firing rate $f^{\text {m}}$ was taken as 7Hz (Effect of motor 1989). The recruitment thresholds were adjusted such that approximately 50% of motor units were recruited for a 20% force level (Scutter and Türker 1998). Lastly, we computed an equivalent, spatially constant activity by a weighted sum of individual motor unit activity and the total normalised motor unit volume fraction of distribution $n=1$ (Fig. 2b).

*(Step 3)* To simulate a static bite, i.e. contraction of the masseters does not displace the mandible, fixation boundary conditions were applied to the molar occlusal surface and the left and right fossas. The articular discs were fixated relative to the mandibular condyles and assumed to be in frictionless sliding contact with the fossas. The masseters were attached to the mandible over manually defined attachment areas and were fixated at their origins. The attachment areas extended caudally from approximately the middle of the ramus to the angle of the mandible. The molar was fixated over its occlusal surface, over which bite force was measured.

We recorded the bite force magnitude along the left-right, posterior-anterior and caudal-cranial axes at the right molar. Additionally, the angles made by the bite force vector with the horizontal and vertical planes were recorded. Simulations were performed using 32 cores (AMD Opteron-6373 @ 2.3 GHz) with 24 GB memory.

#### Motor unit distribution metrics

To quantify each $n=1,\ldots ,7$ motor unit territory distribution, three metrics were computed: the Motor Unit Neighbour Index (MUNI), the Motor Unit Territory Area (MUTA), and the mean active territory distance from the muscle centre of mass ($d_{\text {c}}$). Note that these metrics were computed prior to homogenisation within the finite element mesh and representative metrics were taken from the right masseter. We computed the MUNI as follows: five random virtual tissue samples (dimensions: $5\,\mathrm{mm}\times 5\,\mathrm{mm}$) were taken from the right masseter cross section, and the number of unique motor units within each tissue sample were counted. These values were then averaged to compute MUNI for each distribution. We computed MUTA for each motor unit, i.e. MUTA$^n_i$ ($n=1,\ldots ,7$, $i={1,\ldots ,N_{\text {MU}}}$), as the area of the convex hull over the fibres of a motor unit territory divided by the total cross-sectional area of the masseter (626 mm$^2$). Lastly, $d_{\text {c}}^n$ was computed as the distance between the barycentre of the active motor units and the masseter centre of mass at the initial time step.

## Results

### Mesh dependency of muscle activity

**Fig. 3:**
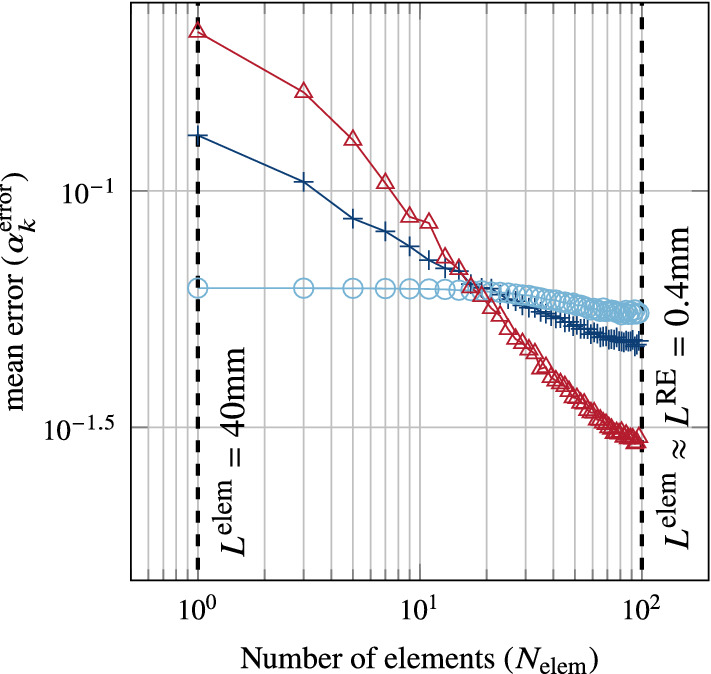
Mesh dependency of the spatial distribution of muscle activity in the idealised mesh. The error is the mean difference between muscle activity at the reference element (RE) and mesh elements. The three curves represent different degrees of motor unit overlap; circular marks for maximum overlap (random distribution), plus marks for medium overlap, and triangle marks for minimal overlap. The vertical dotted lines show the possible extremes of mesh size

The mean error in muscle activity (Eq. 16) for the 3 motor unit distributions (maximum, medium, and minimal overlap) is plotted against mesh size in Fig. 3. At the coarsest mesh size, i.e. 1 element, the mean errors were 0.062, 0.13, and 0.22 as motor unit overlap decreased. Conversely, for the finest mesh size, i.e. when the element size is close to the reference element (RE) size, the means errors were 0.055, 0.047, and 0.030 as motor unit overlap decreased. The degree of mesh dependency was inversely proportional to the degree of overlap.

### Influence of action potential propagation on force production

**Fig. 4:**
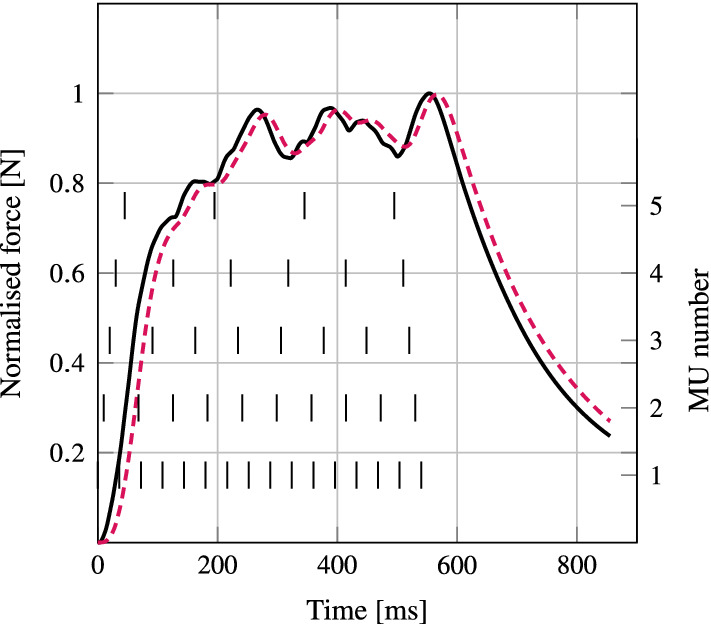
Tendon force of the idealised geometry for the model with action potential propagation (dotted, red) and without action potential propagation (solid, black). The motor unit firing times are indicated by the vertical ticks for each motor unit (right axis)

Computational times for the case with and without action potential propagation were 12 h and 6 h, respectively. The reaction forces for both cases, summed over the nodes at the fixed tendon end, are shown in Fig. 4. The force response for the action potential propagation case is slightly smoother and delayed in time compared to the non-propagation case. The difference in time to force between both models was 16.6ms. Both models reached the same maximum force magnitude of 39N. The electromechanical delay for the cases with and without action potential were 18.8 ms and 8.2 ms, respectively.

### Masseter motor unit anatomy

**Table 2:** Force magnitude ($F_{\text {M}}$) and horizontal orientation ($\theta _{\text {H}}$) at point of peak force production. Motor unit territory metrics: Motor Unit Neighbour Index (MUNI), Motor Unit Territory Area (MUTA) (masseter cross-sectional area 626 mm$^2$), and centre of contraction $d_{\text {c}}$ are also shown for each distribution. The subscript 10 and 90 refer to the 10th and 90th percentile of the MUTAs. Distribution “const.” refers to the status quo, spatially constant activation model

Dist. (n)	$F_{\text {M}}$ [N]	$\theta _{\text {H}}$ [degree]	$d_{\text {c}}$ [mm]	MUNI	MUTA$_{10}$ (%)	MUTA$_{90}$ (%)
const	146.7	− 70	0.0	50	100	100
1	149.1	− 114	6.1	6.70	1	6
2	148.6	− 100	6.1	15.6	8	29
3	144.6	− 76	0.4	7.70	1	13
4	148.0	− 86	4.4	18.4	13	40
5	145.5	− 76	0.7	7.10	1	11
6	144.5	− 74	0.8	18.1	5	43
7	143.8	− 78	2.2	13.3	4	24

**Fig. 5:**
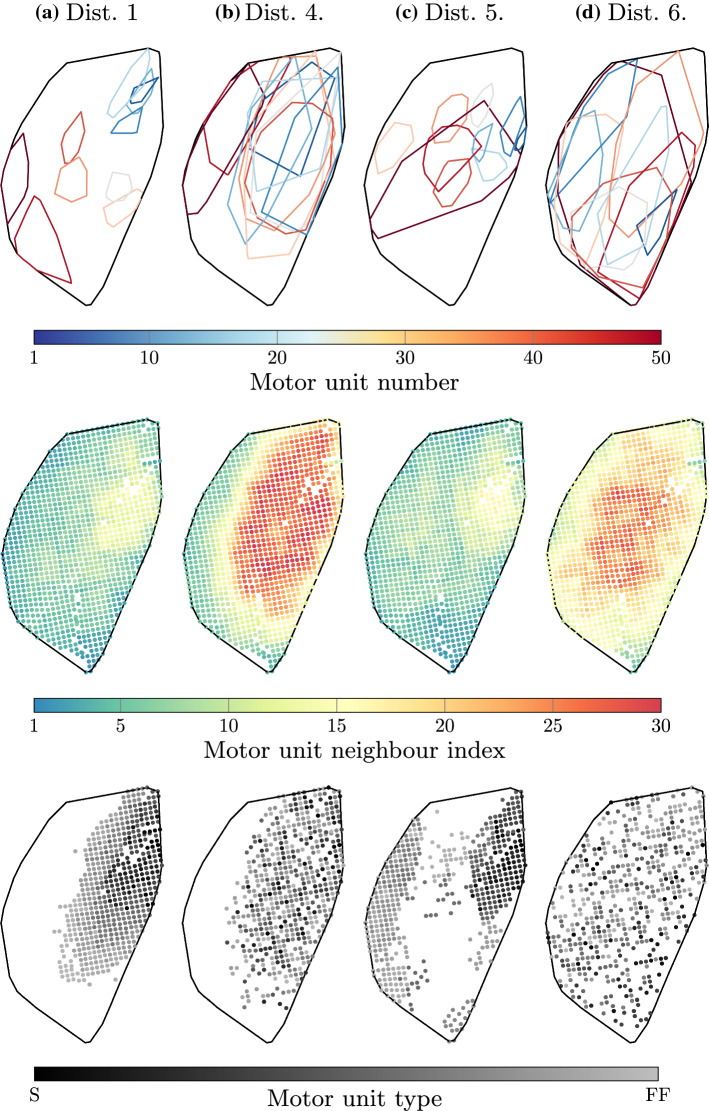
Discrete neuromuscular anatomy over the left masseter cross section in the horizontal plane, at approximately the middle of the muscle. Each mark represents a discrete fibre. Each figure’s top, bottom, left and right corresponds to the anterior, posterior, lateral and medial parts of the muscle, respectively. The top row shows selected motor unit territories. The middle row shows the motor unit neighbour index (MUNI) of each fibre. Any gaps here are visualisation artefacts. The bottom row shows the type of motor unit, where S and FF refer to slow and fast fatiguing motor units, respectively. Only those motor units which are active during the static bite are shown for clarity, i.e. the white spaces are inactive

Examples of the virtual neuromuscular anatomy are shown over the masseter cross section in Fig. 5. Further, the quantitative motor unit metrics for all motor unit distributions are given in Table 2.

For small reference seed point search parameters $\{D, \Delta D\}$, motor unit types were confined to subregions of the masseter cross section. For example, distributions 1, 4 and 5 ($\{D, \Delta D\}=\{ 10\,\mathrm{mm},2\,\mathrm{mm}\}$) show type-S (smaller) motor units positioned anterio-medially in the masseter (Fig. 5). On the other hand, larger values $\{D, \Delta D\}=${300 mm,2 mm} resulted in scattered motor unit territory (distribution 6 in the aforementioned figure).

For larger motor unit axon search parameters $\{R, \Delta R\}$, more overlap was observed between neighbouring motor unit territory. For example, distribution 4 shows a larger overlap than distribution 1 (Fig. 5). This is also reflected in the motor unit territory features. That is, larger values of $\{R, \Delta R\}$ resulted in larger values of Motor Unit Neighbour Index (MUNI) and Motor Unit Territory Area (MUTA) (Table 2). For example, MUNI values of 35.2 and 34.4 (distributions 4 and 6) corresponded to $\{R,\Delta R\}=\{1\,\mathrm{mm},20\,\mathrm{mm}\}$ and $\{R,\Delta R\}=\{15\,\mathrm{mm},4\,\mathrm{mm}\}$, respectively. Conversely, the MUNI values of 12.2 and 11.2 (distributions 1 and 5) corresponded to $\{R,\Delta R\}=\{1\,\mathrm{mm},4\,\mathrm{mm}\}$, yielding more segregated motor units. The reference seed point search parameters $\{D, \Delta D\}$ influenced MUNI to a lesser degree. A similar relationship between the distribution parameters and MUTA was observed. That is, the axon search parameters $\{R, \Delta R\}$ correlated (positively) to a much higher degree to MUTA than did $\{D, \Delta D\}$.

Note that for the spatially constant activity case, each of the 50 motor units exists at all points. Therefore, MUNI$^{\text {const}}=50$, and since each motor unit territory covers the entire muscle, MUTA$^{\text {const}}$ for each territory and the cross-sectional muscle area are identical; at 100 %.

### Bite force specificity of masseters

Computational times for all static bite simulations were between 10 h and 15 h. Cross-sectional masseter activity at point of maximum force production is shown for selected distributions in Fig. 7. The cross sections reveal heterogeneous activity, which differs among the distributions e.g. distributions 1, 4 and 5. The differences in masseter activity patterns also produce different bite forces. The horizontal bite force component at the corresponding time instance is overlaid in Fig. 7.

The distance between the barycentre of contraction and the masseter centre of mass $d_{\text {c}}$ is given in Table 2. Smaller $\{D, \Delta D\}$ resulted in larger $d_{\text {c}}$ values (4.4–6.1 mm) (distributions 1,2 and 4), meaning the barycentre of contraction was positioned further away from the masseter centre of mass. Conversely, as $\{D, \Delta D\}$ increased, $d_{\text {c}}$ tended towards zero, e.g. distributions 3, 5 and 6 all have $d_{\text {c}}<1\,\mathrm{mm}$.

**Fig. 6:**
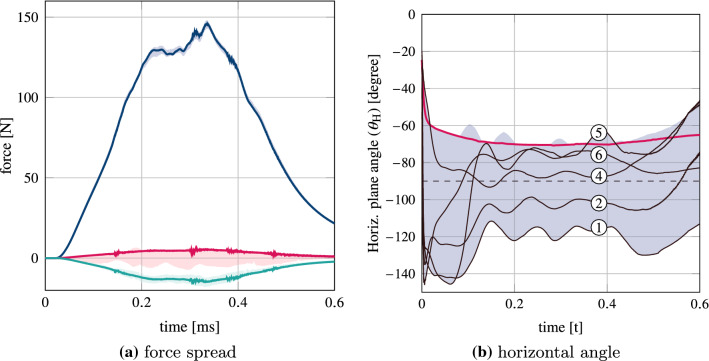
Bite force (**a)** magnitude and (**b)** horizontal angle during the sub-maximal static bite. Shaded regions show the spread from the motor unit driven models. The thicker, solid traces represent the status quo, spatially constant activation model. In (**b)** the thinner, black traces are from selected motor unit driven models, with distribution numbers shown above the trace. The dotted line at − 90$^\circ$ represents the left-right axis

The evolution of the bite force components along the left-right, posterior-anterior and caudal-cranial axes, respectively: $F_{\text {LR}}^n$, $F_{\text {PA}}^n$ and $F_{\text {CC}}^n$, for $n=1,\ldots ,7$ distributions, are plotted in Fig. 6, together with the force components of the spatially constant activity model. The peak force magnitude for the spatially constant activity model was $F_{\text {M}}^{\text {const}}= 147 \mathrm{N}$. The peak force magnitudes for the $n=1,\ldots ,7$ distributions had a mean and range of 146 N and 5 N, respectively. The peak forces in the left-right, posterior-anterior and caudal-cranial axes had means of 14N, 1 N, and 145 N, respectively. The corresponding ranges were 6N, 4 N, and 6 N.

The evolution of the angle of the horizontal bite force component for the $n=1,\ldots ,7$ distributions is shown in Fig. 6b, together with the horizontal angle for the spatially constant activity model. At the point of maximum force, the horizontal angle for the spatially constant activity model was $\theta _{\text {M}}^{\text {const}}= -70^\circ$ (note that 0 $^\circ$ corresponds to the anterior direction and − 180$^\circ$ to the posterior direction). The horizontal angles for the $n=1,\ldots ,7$ distributions had a mean and range of − 86$^\circ$–40$^\circ$, respectively. For the spatially constant activity model, the force was oriented anteriorly and showed minimal variation during the (ramp) contraction. In general, the motor unit driven models produced bite-forces which were less steady in their horizontal angle, with some models even showing a reversal in bite-force direction along the posterior-anterior axis, i.e. distributions 5 and 6. Bite forces of distributions 1 and 2 were oriented posteriorly during the entire contraction, and distributions 3, 7, and 4 were oriented anteriorly during the entire contraction.

**Fig. 7:**
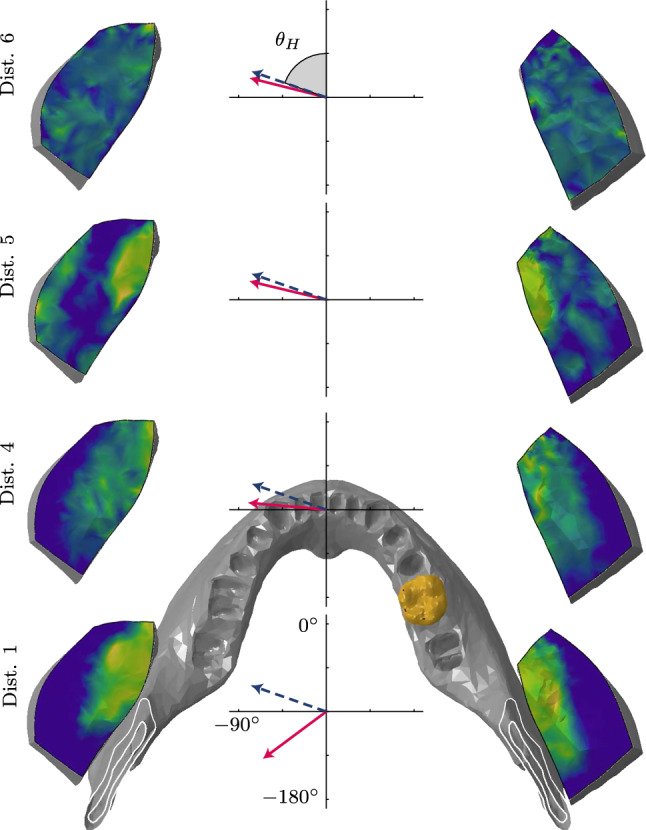
Cross sections of masticatory models (approximately at the mid-point of the muscle), each with a different motor unit territory distribution, taken at the point of maximum force production $t=0.34 \mathrm{s}$. Muscle activity is colour-coded between blue ($\alpha _{\text {m}}=0$) and bright yellow ($\alpha _{\text {m}}=1$). Plots of the bite force component in the horizontal plane, for the corresponding distribution, are also shown, with the dashed blue line representing the status quo, spatially constant activation model and the solid red line representing the motor unit driven model

## Discussions

In some regards, continuum-mechanical models of skeletal muscles (3D models) assume a lumped function. For example, a (single) representative muscle fibre is used to characterise the entire muscle’s activity level. However, muscle contractile properties and activity are heterogeneous due to the muscle’s organisation in motor units. This means that, the selective recruitment of regionally confined motor units can allow task specific muscle activations. Here, we presented a method to introduce such heterogeneity within 3D models by incorporating motor unit distribution and activity information. While this incurred minimal additional computational costs, it did increase model complexity. The question then arises: when is the inclusion of motor unit information in 3D models paramount to muscle motor output predictions? We address this question in regard to motor unit distribution and functional heterogeneity of muscles. Having shown the conditions under which such a motor unit enriched 3D model is beneficial, we conclude with the limitations of the current model and its implications for continuum-mechanical muscle models.

### Motor unit distribution and bite force

Muscles contain hundreds to thousands of motor units, and motor units, in turn, contain tens to thousands of muscle fibres, and represent the smallest unit that can be controlled individually by the nervous system. A motor unit’s fibres are often regionally confined within the muscle. For example, higher numbers of small motor units were found deep in the masseter (Eriksson and Thornell 1983; Tonndorf and Hannam 1994; van Eijden and Turkawski 2001), or superficially in the tibialis anterior (Mesin et al. 2010). The sequential recruitment of locally confined motor units results in localised muscle contraction. Accordingly, fibre orientation within this region dictates the force vector produced. For example, Turkawski et al. (1998) found, by individually recruiting motor units, that the rabbit masseter produced “lines of action ... at least as large as the variation in fibre directions”. Other studies show a similar link between regional muscle (or motor unit) activity and changes in force vector (Schindler et al. 2005; Ogawa et al. 2006; Holtermann et al. 2009; Miyamoto et al. 2012).

This work presents a computational model to demonstrate the relationship between motor unit anatomy, recruitment, and bite force using anatomically realistic masticatory model. The results showed that bite force magnitude was mildly affected upon variation of recruitment regions (range of 5 N, for an average 146 N); however, significant differences were observed in bite force direction (range of 40$^\circ$, for an average of − 86$^\circ$). Furthermore, differences in force direction seemed to increase as the motor unit territories were less overlapped. For example, distributions 1 and 5 had highly segregated motor units (motor unit neighbour index (MUNI) of 6.7 and 7.1, respectively) and produced a highly oscillating force direction compared to distributions 4 and 2 (MUNI of 18.4 and 16.6, respectively) (Table 2 and Fig. 5). Recalling that high MUNI values correspond to increased motor unit overlap. As motor units become more overlapped, their recruitment results in the excitation of the same muscle region, therefore producing a more stable force direction. Indeed, as MUNI values increased, from 6.7 to 15.6 to 18.4 (distributions 1, 2 and 4, respectively), the steadiness of bite force direction increased (Fig. 6b). Taken to the extreme, all motor units would overlap at every point within the muscle; this is the assumption behind the spatially constant activity case (which has a theoretical MUNI of 50). As expected, this produces the most steady force direction (Fig. 6b).

The link between motor unit overlap and force direction reveals the interplay of muscle architecture and neural organisation. That is, a muscle’s force direction can only be modulated by the nervous system when fibres of the motor units are regionally confined. The masseter has more confined territories as compared to muscles of the limbs —“suggest[ing] a more localised organisation of motor control in masticatory muscles” ( van Eijden and Turkawski 2001). Additionally, Schindler et al. (2014) found that small “sub-volumes” of the masseter were recruited under particular tasks via the recruitment of adjacent motor units. This suggests that modelling masseter activity (and that of other muscles with complex architecture) as spatially constant may oversimplify force predictions.

In these static bite simulations, masseter architecture explains the relationship between motor unit overlap/segregation and force (direction) steadiness. The masseters were defined as having a smaller deep head and a much larger superficial head. The deep (or inferior) head arises from the deep surface of the zygomatic arch and inserts on the upper part of the ramus. The superficial (or superior) head is much thicker and arises from the anterior two-thirds of the zygomatic arch and inserts to the angle of the mandible. When viewed from the sagittal plane, fibres of the deep and superficial heads form an “x”, i.e. they are aligned posterio-cranially and anterio-cranially, respectively. In the bite simulation, the same set of (34 out of 50) motor units were recruited via identical firing patterns. The only difference was the distribution of their fibres within the masseters (Fig. 5, bottom row). When coupled with the different fibre directions in the deep and superficial head, this means that the relative location of the contracting fibres influences the line-of-action. Figure 7 shows the pattern of activity over cross sections of the left and right masseters, at the point of maximum recruitment —for different motor unit distributions. The activity patterns correspond well with the motor unit fibre distributions in Fig. 5 (bottom row), as expected. The horizontal bite force angle is also shown in Fig. 7, and reveals that as the recruited fibres become more dispersed throughout the masseter (from distribution 1 to 6), the force direction converges to the spatially constant activation case. More specifically, distributions 1 and 2 had contracting motor units in the deep head, and thus the bite force was directed posteriorly (horizontal angles of − 114$^\circ$ and − 100$^\circ$, Table 2). Note that a horizontal angle of 0$^\circ$, − 90$^\circ$, and − 180$^\circ$ refers to forces directed anteriorly, to the left, and posteriorly, respectively (Fig. 7). Distributions 3, 5, 6, and 7 produced anteriorly directed bite forces (horizontal angles between 70$^\circ$ and 80$^\circ$) since most of the contracting motor units were located in the superficial head. Interestingly, distributions 5 and 6 produced a bite force that switched direction; posteriorly to anteriorly. Here, motor units are initially recruited from the deep head and later from those located in the superficial head.

These findings are in agreement with experimental observations, which show the involvement of the masseter’s deep and superficial regions to retract and protrude the mandible, respectively. For example, Belser and Hannam (1986) found that during jaw retraction, the respective activity of superior and deep fibres was 5.5 % and 47.5 %. Additionally, Hannam and McMillan (1994); Blanksma et al. (1997) found that deep fibres in the masseter contributed predominantly to jaw elevation and jaw retraction. Recording the activity of individual motor units, Ogawa et al. (2006) found that those motor units located deep within the masseter started firing earlier when the bite was oriented “posterio-laterally”, i.e. retraction of the jaw laterally. As the bite force became more anterior, the motor units in the superficial region became more active.

Although distributions 5 and 6 produce similarly oriented bite forces, the motor unit territory in distribution 6 show much greater overlap than distribution 5. This implies that motor unit territory overlap alone does not characterise the force behaviour; rather the position of the motor unit territory is of paramount importance. This is reflected by the fact that distributions 5 and 6 have a similar mean barycentre of contraction ($d_{\text {c}}$ in Table 2), again, despite having differing amounts of territory overlap. Conversely, distributions 2 and 7 have similar amounts of overlap and territory areas; however, since they differ in their mean barycentre of contraction, one produces a slightly higher force, oriented posterio-cranially (distribution 2), while the other is oriented anterio-cranially. Subtleties of muscle function as discussed above cannot be captured without taking motor unit distributions into account.

### Assumptions and limitations

Force production within a fibre follows the propagation of the action potential. As with status-quo 3D models, we assume that action potential propagation time (in the order of milliseconds) is negligible when compared to the temporal scale of movements (in the order of seconds). When action potential propagation was ignored, the force detected at the tendons was advanced by approximately 20ms. The geometry and material properties of the musculotendon complex impact this value. For example, Schmid et al. (2019) showed that a low muscle-to-tendon ratio, long fibres, stiff muscles and soft tendons act to (individually) increase electromechanical delay. Therefore, care should be taken when action potential propagation is neglected for muscles fitting this profile.

Muscles produce force by the orderly and sequential recruitment of their motor units. Motor unit recruitment may, however, be altered in some cases, such as the task performed, contraction velocity, and sensory feedback (see Hodson-Tole and Wakeling 2009, for example). Alternate recruitment strategies may enhance regional contractions since the nervous system could recruit neighbouring motor units. For example, Schindler et al. (2014) found localised task groups of motor units in the masseter. In the current model, motor units are recruited strictly according to the size principle, i.e. modification of recruitment thresholds is not accounted for.

The homogenised continuum-mechanical model does not explicitly resolve the muscle fibres. Instead, the most important properties of a muscle’s fibre architecture, in terms of mechanical force production, are included in our model in a continuous manner. Specifically, the fibre orientation field and the motor unit volume fractions. Similar assumptions have been used in the past for chemo-electro-mechanical, multi-physics models of skeletal muscle tissue. This is mostly due to the fact that models explicitly resolving the full microscopic muscle geometry are typically limited to simulate small tissue samples due to computational costs (Sharafi and Blemker 2011; Virgilio et al. 2015; Spyrou et al. 2017, 2019, e.g). However, a continuous representation of motor unit anatomy introduces mesh dependent model error. The mesh dependency study revealed that the mean error in muscle activity depends on both mesh size and motor unit overlap. For maximal amount of overlap, i.e. randomly distributed motor units, the error did not show convergence with mesh refinement. This is because each element has a very high probability of containing all the muscle’s motor units. Therefore, as element size changes it has little to no effect on the mean activity within the element. On the other hand, minimally overlapped motor units show a strong reduction in error upon refinement. This is because low motor unit overlap leads to regions in the muscle that contain only a single motor unit. Then, as the mesh size decreases, the probability that an element covers a region that contains only a single motor unit increases. This explains the observation that the error for the minimally overlapped case drops below that of the random distribution case, since random motor unit distribution will not yield elements that contain a single motor unit. Regarding the proof-of-concept masticatory simulation, the element edge lengths in the masseter ranged between $\approx 2-3\, \mathrm{mm}$, which corresponds to roughly 6 % mean error in the computed muscle activity. It should be noted that mesh size does not influence muscle activity alone, but rather also impacts the fibre orientation and stress–strain fields. A comprehensive convergence analysis is an important next step in verifying the proposed method, and finite element skeletal muscle models in general.

We assumed that fibre size is the same within and between motor units. It has been observed, however, that fibres between motor unit types differ in size (e.g. Polgar et al. 1973) and have normally distributed diameters within a motor unit type (e.g. Hegarty and Hooper 1971). Furthermore, contractile properties also seem to vary between motor unit types. Kanda and Hashizume (1992) showed, first, that mean muscle fibre cross-sectional area increased proportionally with innervation ratio. Second, as innervation ratio increased, so did the specific tension (motor unit tension divided by motor unit cross-sectional area area). Therefore, not only do larger motor units occupy more space, but they produce a greater force per unit of area. Factors which govern the peak tension of a motor unit in the current model are activation, specific strength, and innervation ratio. The activity of each motor unit is normalised (Eq. 7) between [0, 1] and the specific strength is not specified per fibre, but rather by the peak isometric stress, i.e. $S_{\text {max}}$, and is constant over the entire muscle (Eq. 4). Therefore, neglecting geometrical effects and assuming full activation of all motor units, differences in peak force between motor units depend solely on innervation ratio. While the inclusion of these motor unit characteristics would yield more accurate motor unit force production, it appears that the innervation ratio of a motor unit is the dominant factor in predicting its force output (Kanda and Hashizume 1992). Therefore, from a functional point of view, although fibre diameters and specific tension are idealised, the relative motor unit force contribution is maintained in the current model.

### Implications

The interplay of motor unit anatomy, selective recruitment by the nervous system, and the architecture of the muscle gives rise to regional contraction and functional heterogeneity of the muscle. From a theoretical point of view, this would be most apparent for muscles with “complex” architecture (bipennate or multipennate) and those with a large number of segregated motor units. We hypothesise that the more versatile a muscle needs to be, the more complex its architecture and the more segregated its motor units. This is intuitive since a complex architecture can only be exploited by the nervous system if the motor units are regionally confined.

Joint kinematics typically result from the coordination of multiple spanning muscles. One way to determine a muscle’s contribution to a movement is via inverse kinematics and optimisation techniques. Typically, modelling studies assume each muscle to operate as a single contractile unit, whether using 1D Hill-type models or 3D models (Péan et al. 2019; Harandi et al. 2020). However, by considering the joint as spanned by groups of motor units instead, the spatial resolution of force production would be greatly increased. Consequently, the sub-maximal contraction of motor units would no longer simply scale the resultant force vector’s magnitude but would also influence its direction and thus the joint torques produced (as shown by the bite force in the present study). This has further implications for muscle function and coordination, such as fatigue during sustained contractions. Here, the coordination of motor units across multiple muscles could be optimised in such a way as to maintain the posture, e.g. via recruitment strategies such as motor unit substitution or rotation (Bawa and Murnaghan 2009).

## Conclusions

In this paper, we developed a method to generate and include motor unit activity and anatomical information in 3D continuum-mechanical muscle models (3D models). Although idealised, the proof-of-concept simulation study showed that enriching 3D models with motor unit information significantly impacted bite force. In summary, (i) activating different regions of the masseter had a minimal effect on bite force magnitude, but affected bite force direction significantly; (ii) activation of the deep and superficial regions of the masseter yielded a more posteriorly and anteriorly directed bite force, respectively; (iii) lower overlap between motor units yielded a higher variation in bite force direction; and (iv) motor unit overlap has a smaller impact on bite force (magnitude and direction) than motor unit position. These findings suggest that the current modelling approach would benefit muscles with complex internal architecture and segregated motor units. The proposed method was shown to reasonably approximate skeletal muscle physiology. Nevertheless, a rigorous validation is still required. In particular, the in vivo characterisation of motor unit territories presents a unique challenge. Despite this, the present modelling framework enables, at least in a qualitative sense, the investigation of motor unit properties within the context of muscle motor output and joint function. This is relevant not only for understanding the functional heterogeneity of healthy muscle but also for aged or pathological muscle, where motor unit remodelling takes place.

## Supplementary Information

Below is the link to the electronic supplementary material.

Supplementary file 1 (pdf 1084 KB)

## Data Availability

Not applicable

## Footnotes

1 Muscle activity at various mesh sizes for the 3 motor unit distributions covered are plotted in the supplementary material.
