# Supplementary figures and images for: Modelling motor units in 3D: influence on muscle contraction and joint force via a proof of concept simulation
Source: Biomech Model Mechanobiol. 2022 Dec 27;22(2):593–610. doi: 10.1007/s10237-022-01666-2 (PMC10097764; doi:10.1007/s10237-022-01666-2)

Maximum overlap (random distribution)

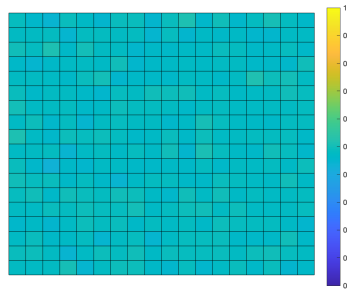

Mesh size: 2.1mm

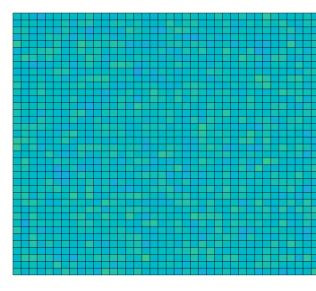

1.0mm

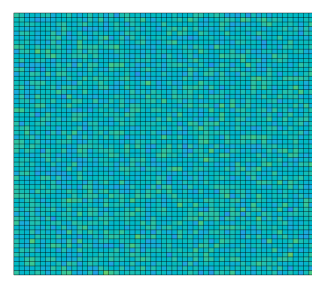

0.7mm

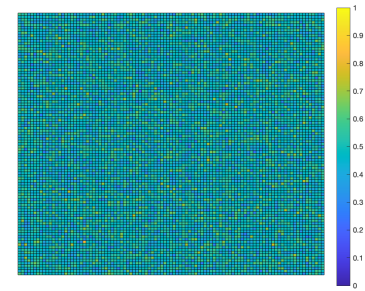

0.4mm

Medium overlap

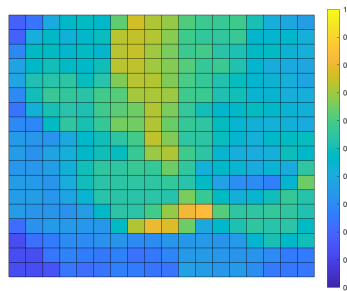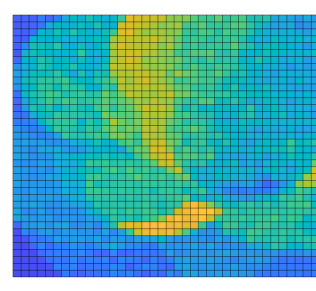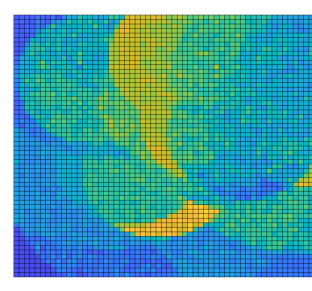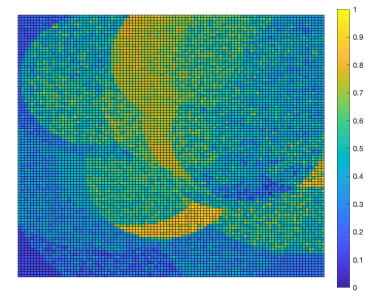

Minimum overlap

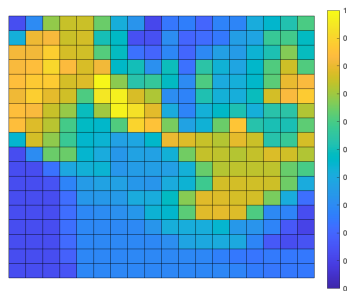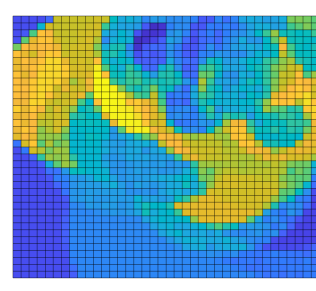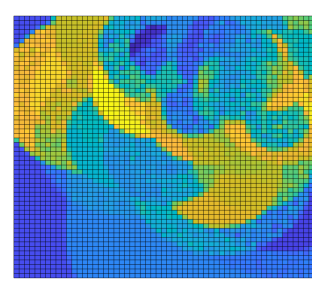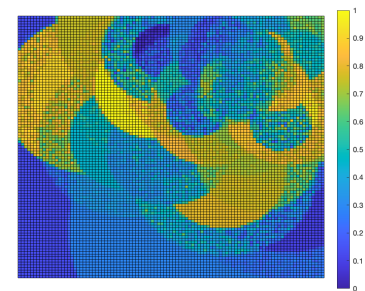

Supplement: Supplementary file 1 — (pdf 1084 KB) [file 10237_2022_1666_MOESM1_ESM.pdf]
